# The Essential Involvement of the Omentum in the Peritoneal Defensive Mechanisms During Intra-Abdominal Sepsis

**DOI:** 10.3389/fimmu.2021.631609

**Published:** 2021-03-18

**Authors:** Ying Liu, Jian-nan Hu, Ning Luo, Jie Zhao, Shu-chang Liu, Tao Ma, Yong-ming Yao

**Affiliations:** ^1^ Department of General Surgery, Tianjin Medical University General Hospital, Tianjin, China; ^2^ Department of Intensive Care Unit, Tianjin Medical University General Hospital, Tianjin, China; ^3^ Department of Microbiology and Immunology, Trauma Research Center, Fourth Medical Center of the Chinese PLA General Hospital, Beijing, China

**Keywords:** omentum, milky spots, peritonitis, B1 cells, bacterial clearance

## Abstract

Although the abilities of the omentum to alleviate inflammation and prevent infection have been revealed over the past decades, the underlying mechanisms remain largely unelucidated. Here, we demonstrated that the mortality of mice exposed to cecal ligation and puncture (CLP) and omentectomy was remarkably increased compared to those treated with CLP alone. Moreover, the efficacy of the omentum was associated with an impairment in intraperitoneal bacterial clearance together with an increase in the expression of proinflammatory cytokines. Besides, in response to peritoneal infections, the size and quantity of the omental milky spots (MSs) were increased tremendously and they also support innate-like B1 cell responses and local IgM production in the peritoneal cavity. Furthermore, not only the migration but also the functional activities of neutrophils were diminished in the absence of the omentum. These data collectively show that the omentum contributes more to peritoneal immune responses during septic peritonitis than has heretofore been recognized. Thus, harnessing the function of MS-containing omentum to increase its protective effectiveness may exert important biological and therapeutic implications for the control of intra-abdominal infections.

## Introduction

Peritonitis is induced by multiple clinical conditions such as perforation of a hollow viscus, anastomotic leakage of the gastrointestinal tract and penetrating infectious or cancerous processes, which is usually life-threatening (1). During peritonitis, contamination of the peritoneal cavity can lead to an infection, thus initiating the inflammatory responses in an attempt to expel pathogens. If the infection is unable to be eradicated, a life-threatening systemic insult with high mortality rate may be induced by intra-abdominal sepsis (2). Despite recent improvements in surgical techniques and intensive care management, the death rate attributable to secondary peritonitis accounts for ~30% (3). Until now, the development and progression of intra-abdominal sepsis remain largely unclarified. It has been well known that sepsis is a multifactorial condition regulated by various factors such as the initial injury, responsiveness of the body, age, gender and genetic background of the patient (4). Hence, the identification and understanding of the defensive responses to peritoneal infections are of great importance for discovering effective interventions and therapeutic approaches (4).

As an intraperitoneal organ, the greater omentum or omentum majus is composed of two layers of mesothelial tissues that encapsulate a high density of parenchymal adipose cells and immune cell clusters or “milky spots” (MSs) (2). These MSs actively gather fluids, transfer particulate matter from the peritoneal cavity, occur before immunization and exhibit antigen-specific CD4 and CD8 T cell as well as B cell responses. Thus, MSs have been recognized as secondary lymphoid organs, despite that it’s structure, development and functionality are unique (5, 6). MSs are highly vascularized and wrapped around the glomerular capillaries. Some of these capillaries contain high endothelial venules, which is essential for immune cell trafficking (7). Additionally, the cell populations in MSs are also unique, in which B1 cells, proficient in providing crucial IgM antibodies for pathogen clearance, make up the majority of lymphocytes (8). Importantly, owning to the fact that it can alleviate peritoneal inflammation and enhance wound recovery, the omentum has been known as an abdominal policeman (7). Previous clinical research demonstrated that the incidence rate of patients with peritonitis was increased after excision of the omentum (4); while an experimental study also showed that removal of the omentum could reduce survival rate and affect certain defense mechanisms (2). Although the excellent abilities of the omentum for alleviating inflammation and preventing infection have long been appreciated by surgeons, the mechanisms underlying its anti-inflammatory and anti-microbial effects remain largely unclarified.

A rapid, orchestrated phagocytic response is indispensable for the successful resolution of peritoneal infectious insult. In general, septic peritonitis is characterized by a massive infiltration of neutrophils into the peritoneal cavity, where they express potent antimicrobial activity such as phagocytosis, reactive oxygen species, cytokines, proteases and chemokines, and their infiltration is important for the clearance of bacteria from the peritoneal cavity (9–11). Recently, omental MSs have been demonstrated to be the main entry pathway for the recruitment of neutrophils to the peritoneal cavity during peritonitis (2). Besides, in view of their abilities to collect antigens, particles and pathogens from the peritoneal cavity, MSs have been recognized to resemble the secondary lymphoid follicles and contribute to peritoneal immunity depending on the stimuli (12). Then, in the present study, we first verified the potential roles of omental MSs in mediating the peritoneal immune response provoked by CLP-induced peritonitis, and further provided definitive evidence that the excision of omental MSs could diminish B1 cell responses, attenuate IgM production and impair neutrophils migration and function in the peritoneal cavity, which consequently leads to systemic infection and exacerbating sepsis. Thus, harnessing the function of omental MSs may have important biological and therapeutic implications for the effective control of peritoneal infections.

## Materials and Methods

### Animals

Wild-type (WT) C57BL/6 mice were purchased from Beijing Vital River Laboratory Animal Technology Co. Throughout this study, all mice were used at 8-10 week-old with 22-28 g body weight. All animals were housed under specific pathogen-free facilities and fed a standard laboratory diet. Before the initiation, all experimental manipulations were performed in accordance with the National Institute of Health Guide for the Care and Use of Laboratory Animals, with the approval of the Scientific Investigation Board at the Tianjin Medical University.

### CLP

As previously described, cecal ligation and puncture(CLP) surgery was performed to induce sepsis (13). Mice were anesthetized deeply with isoflurane. Under sterile conditions, a 2-cm-long midline cut was made at the abdomen region to expose the cecum. At a point ∼1 cm from the cecal tip, the cecum was ligated with a 4-0 silk suture and punctured with a 20-gauge needle. The ligated and punctured cecum was squeezed to place a small portion of its contents into the peritoneum and replaced to the abdominal cavity. The incision was sutured with silk sutures in two layers. Thereafter, mice were injected with a 1 mL sterile saline (0.9%) into the scruff of the neck subcutaneously and warmed on a thermal blanket until they recovered.

### Omentectomy

For the surgical removal of the omentum, mice were anesthetized as described above and secured in the supine position. Under sterile conditions, a 2-cm incision was made at the abdomen region to expose the greater curvature of the stomach and omentum. Both most lateral ends of the omentum were ligated to avoid bleeding, then the omentum was removed carefully. After then, the stomach was replaced into the abdomen and this procedure was followed with CLP surgery.

### Confocal Microscopy

After dissection, the whole omentum tissues were fixed in PBS 4% Paraformaldehyde for 1 hour, blocked with 1% bovine serum albumin for 30 min, and then stained with antibodies overnight at 4°C. Antibodies used are as below: Alexa Fluor 488-anti-CD45, Alexa Flour 405-anti-CD11b, eFluor 570-anti-IgM, and eFluor 660 -anti-CD3. Whole-mount tissues were washed twice, each lasting for 30 min with ice-cold PBS, and were mounted with Mowiol. Confocal images were acquired by using a Zeiss LSM 800 confocal microscope with a Hamamatsu ORCA–ER CCD camera and analyzed by Zen software.

### Cells Isolation From the Peritoneal Cavity and Omentum

Peritoneal cells were collected by washout of the peritoneal cavity. Briefly, the peritoneal cavity of mice was injected with 5 mL of ice-cold PBS and massaged gently to dislodge any loosely attached cells. Then peritoneal lavage fluid (PLF) was extracted and washed twice with PBS by centrifugation at 300 × g for 10 min at 4°C. And the resulting pellet was suspended in 1ml ice-cold PBS. After dissection, omentum was washed with PBS, enzymatically digested in RPMI 1640 containing 1% FBS and 1.8 mg/ml Collagenase D (Roche) for 45 min at 37°C. Before staining, tissue suspensions were meshed by using a 70-mm strainer. Then, cells were washed twice with PBS containing 3% FBS and suspended in 1ml ice-cold PBS.

### Flow Cytometry

Cells present in the PLF or obtained from omentum were pretreated on ice for 20 min with anti-mouse CD16/32 abs to block FC receptors. For cell surface staining, cells were stained on ice for 30 min with the following fluorescently conjugated antibodies: peridinin-Chlorophyll-Protein Complex (PerCP)-anti-CD45 (30-F11), phycoerythrin (PE)-anti-CD11b (M1/70), isothiocyanate (FITC)–anti-Ly6G (1A8), PE-anti-CD19 (6D5), and FITC–anti-CD11b(M1/70) (all from BioLegend). Neutrophils were identified by CD11b^+^Ly6G^+^, and B1 cells were identified by CD19^+^CD11b^+^. For intracellular staining, a cytofix/Cytoperm kit (BD) was used to fixed and permeabilized cells. Allophycocyanin (APC)–anti-TNF-α (MP6-XT22) and APC–anti-IL-6 (MP5-20F3) Ab were used for intracellular staining. Isotype controls used were PerCP-conjugated rat IgG2b, PE-conjugated rat IgG2a, FITC-conjugated rat IgG2b, and APC-conjugated rat IgG1 (all from BioLegend). FACS analysis was performed using a BD Accuir C6 Plus cell analyzer (BD). All data were analyzed using FlowJo (Tree Star). For all samples, at least 5×10^4^ cells were collected to generate scatter plots.

### Enzyme-linked Immunosorbent Assay (ELISA)

At the 8 and 24 h after CLP, Whole blood was collected by cardiac puncture using 1-ml syringes (14). To separate serum, blood samples were centrifuged at 1000×g for 15 min. The PLF was obtained by washout of the peritoneal cavity with 5 ml of PBS. To collect the supernatant, PLF samples were centrifuged at 300×g for 15 min. According to the manufacturer’s instructions, using commercial ELISA kits (DAKEWE) to determine the level of TNF-α, IL-6, IL-10, and IgM in the peripheral blood and the peritoneal cavity.

### Bacterial Culture

As described previously, for bacterial culturing, the PLF was obtained by washing the cavity with sterile PBS (5 ml per mice) and the blood was obtained by cardiac puncture (0.5 ml) under sterile conditions (15). The whole blood needs to mix with EDTA (final 4 mM) immediately to prevent coagulation. All the samples were placed at 4°C and diluted in sterile PBS, and 100 μl of each sample was spread on 5% sheep blood Trypticase Soy Agar plates. Plates were incubated in a moist chamber at 37°C for 24 h. The amounts of colonies were counted, and the date expressed as Colony-forming units (CFUs) per milliliter.

### Neutrophils Isolation and In Vitro Phagocytosis Assays

Peritoneal cells were collected as above described. And the collected PLF was filtered to remove debris through a 100-μM filter. Then, neutrophils were isolated by using the Neutrophil Isolation Kit (Miltenyi Biotec). Briefly, resuspended cells were incubated with a neutrophil biotin-antibody cocktail for 10 minutes in the refrigerator (2-8°C), then anti-biotin microbeads were added. After incubating for 15 minutes, the cell suspension was applied to MS column, and flow-through cells were collected. The purity of the collected neutrophils was greater than 90%, as confirmed by using flow cytometry staining with CD11b and Ly6-G antibodies. For *in vitro* phagocytosis assays, isolated neutrophils were incubated with 1×10^6^ CFU of fluorescently labeled E. coli (Abcam) in 24-well culture plates. After 2-hours incubated at 37°C in 5% CO_2_, cells were harvested and washed with PBS to remove unphagocytosed bacteria. For bacterial phagocytosis assay, the FITC-labeled Escherichia coli was measured by flow cytometry. FACS analysis was performed using a BD Accuir C6 Plus cell analyzer (BD). All data were analyzed using FlowJo (Tree Star). For all samples, at least 5×10^4^ cells were collected to generate scatter plots.

### Detection of Reactive Oxygen Species

Cells from peritoneal lavage were plated at 5-10 ×10^5^ cells in 96 well plates and incubated in complete media containing 5 μM/ml ROS Brite™ (10 µM, AAT Bioquest) for 30 minutes at 37 °C. The dye-loading solution was replaced with an HHBS buffer. And then the cells were washed with FACS buffer prior to being stained with the surface markers CD11b and Ly6G to identify neutrophils. FACS analysis was performed using a BD Accuir C6 Plus cell analyzer (BD). All data were analyzed using FlowJo (Tree Star).

### Statistical Analysis

All statistical comparisons were analyzed using GraphPad Prism software (Graph-Pad Software). Statistical significance was analyzed using a 2-tailed t-test. Differences among groups in bacterial CFU were compared using a nonparametric Mann–Whitney U test. Survival curves were analyzed by a log-rank test. All values are presented as mean values ± SEM, except for bacterial counts. For all experiments, a *P-*value of <0.05 was accepted as statistically significant.

## Results

### Increased Susceptibility to CLP Is Associated With Exaggerated Systemic Inflammation in Omentectomized Mice

**Figure 1:**
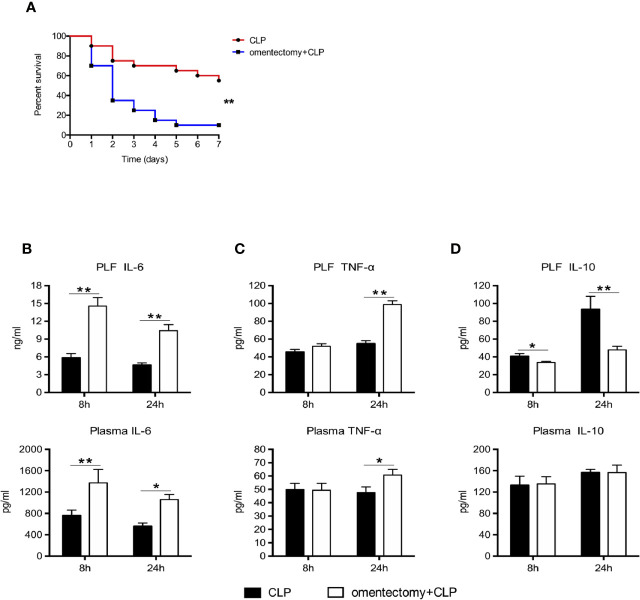
The removal of the omentum aggravates the systemic inflammation and reduces survival after CLP. **(A)** Mice were subjected to CLP or omentectomy+CLP (n = 20 per group). The survival rates were evaluated for consecutive 7 days after CLP. Statistical difference was determined by the log-rank test. At the 8 and 24 h after CLP, PLF and blood samples were collected, and the level of IL-6 **(B)**, TNF-α **(C)**, and IL-10 **(D)** (n = 9~10 per group) were determined. All values are shown as means ± SEM from 3 separate experiments. **p < 0.01 and *p < 0.05 for omentectomized vs WT.

To determine the association between the omentum and susceptibility to CLP-induced peritonitis, we performed CLP procedures on WT and omentectomized mice. Following the insult, the morbidity and mortality rates of mice were recorded continuously. Consistent with previous findings (4), excision of the omentum decreased the mortality rate of mice after CLP surgery. Our data showed that 11 of 20 mice (55%) survived 7 days after CLP, whereas only 10% of mice survived when omentectomy and CLP were performed simultaneously (**Figure 1A**). Considering the possible relationship between inflammation and CLP-induced sepsis, the levels of IL-6, IL-10, and TNF-α in the PLF and blood obtained 8 and 24 h after CLP were measured in order to assess how omentum removal could affect cytokine production in response to infection. The results showed that the levels of IL-6 in the PLF and serum samples obtained at 8 and 24 h after omentectomy and CLP were higher than those exposed to CLP alone (**Figure 1B**). Similarly, higher PLF and plasma levels of TNF-α were detected in the samples obtained at 24 h after omentectomy and CLP when compared with CLP alone (**Figure 1C**). Although there were no significant differences in the plasma levels of IL-10 between the two groups, CLP markedly increased the intraperitoneal contents of IL-10 in WT mice but less so in omentectomized mice. (**Figure 1D**). Collectively, these findings revealed that the absence of the omentum could increase the susceptibility to CLP-induced peritonitis, accompanied by exaggerated systemic inflammatory responses.

### Impaired Bacterial Clearance in Omentectomized Mice

**Figure 2:**
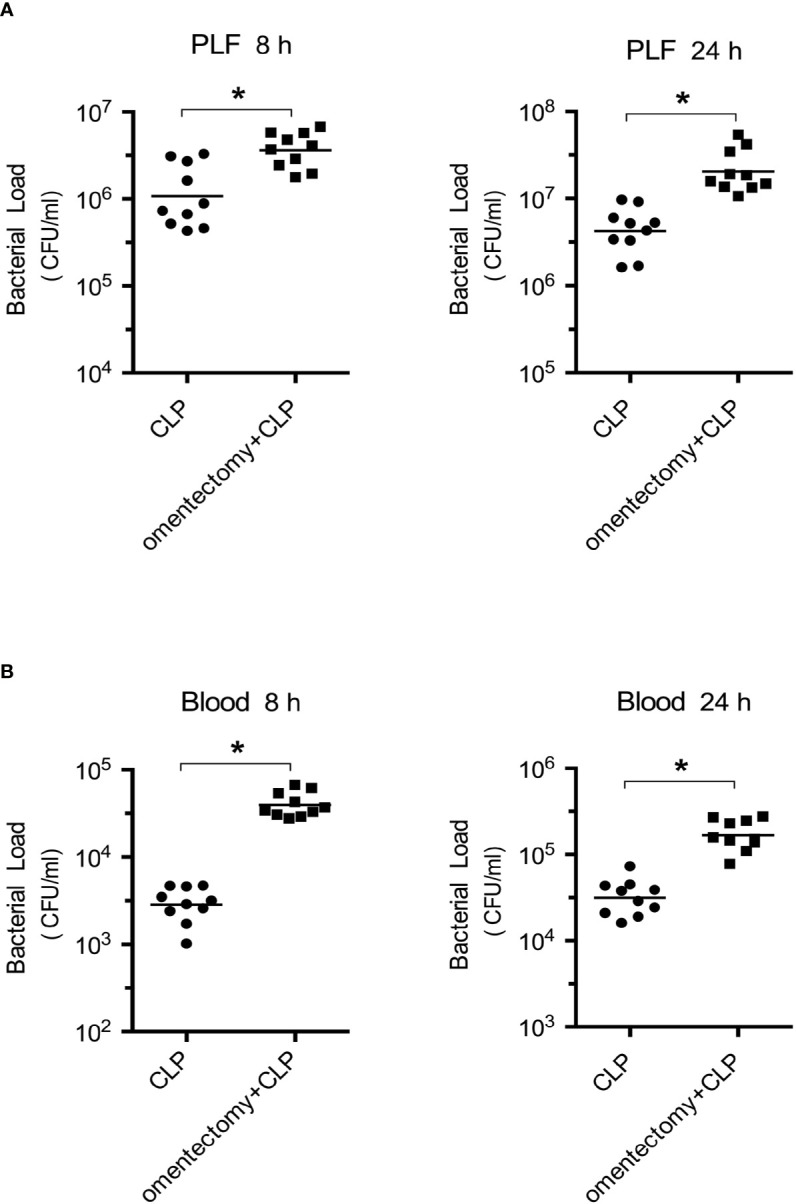
Impaired bacterial clearance in omentectomized mice. At 8 and 24 h after CLP, PLF **(A)**, and peripheral blood **(B)** were collected (n = 9~10 per group). After dilution, 100 μl of each sample was placed on TSA-blood plates, and bacterial CFUs were determined. The horizontal bar represents the mean for each group. Symbols represent individual mice. *p < 0.05 for omentectomized vs WT.

CLP-induced peritonitis can be triggered by the intraperitoneal dissemination of intestinal polymicrobial flora. Hence, to explore whether bacterial clearance can affect the survival of omentectomized mice, the number of CFUs was assessed in the PLF and blood samples obtained from WT and omentectomized mice at 8 and 24 h after CLP surgery (2). Notably, the peritoneal bacteria and blood CFU counts after CLP were markedly higher in omentectomized mice than in WT mice at the indicated intervals (**Figures 2A, B**). These results demonstrated that the omentum exerted protective effects on CLP-induced peritonitis; and in the absence of omentum, bacterial clearance from the peritoneal cavity was impaired, thus resulting in the development of more serious bacteremia and worsen survival.

### Omentum MSs and B1 Cells Are Elicited in Response to Peritoneal Infection

**Figure 3:**
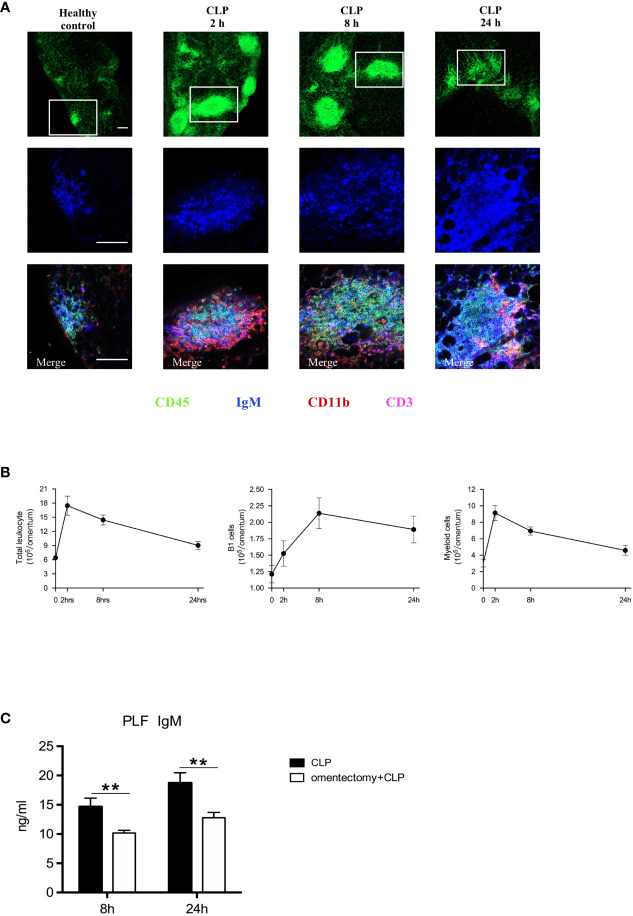
Omental MSs and B cells are elicited in response to peritoneal infections. **(A)** Whole-mount immunofluorescence staining of the omentum allowing visualization of MSs with CD45^+^ staining (green)on the top row, B1 cells with IgM^+^ staining (blue) on the middle row and myeloid cells with CD11b^+^ staining (red), B1 cells with IgM^+^ staining (blue), and T cells with CD3^+^ staining(pink) on the bottom row respectively at indicated time intervals after CLP. Pictures are representative of 6 mice for each condition. The scale bar is 200 μm. **(B)** At the indicated intervals after CLP, flow cytometric analysis of digested omental cells from healthy controls or CLP mice (n = 6 per group) at indicated time intervals were performed and the numbers of total leukocytes, B1 cells, Myeloid cells were counted. All values are shown as means ± SEM. **(C)** At indicated intervals after CLP, PLF samples were collected and IgM concentrations were determined by ELISA. All values are shown as means ± SEM. **p < 0.01 for omentectomized *vs* WT.

MSs in the omentum, which resemble the follicles of secondary lymphoid organs (7), can play a crucial role in regulating peritoneal immunity (16). Consistent with previous findings (12), our data clearly showed that, at the steady-state, relatively small and few MSs were scattered throughout the omentum, and after CLP procedure, the size and quantity of MSs were tremendously increased, particularly during the first 8 h (**Figure 3A**). In healthy controls, MSs were mostly composed of IgM^+^ B1 cells, with low quantities of CD11b^+^ myeloid cells and CD3^+^ T cells. Through the use of whole-mount immunofluorescence staining, we observed a striking increase in the numbers of IgM^+^ B1 cells and CD11b^+^ myeloid cells within MSs after omentectomy and CLP. Moreover, flow cytometric analysis of digested omental cells was also performed at the indicated intervals, and the results indicated that there was a rapid and massive accumulation of immune cells after intra-abdominal infection, which peaked at the first 2-h time point after CLP. Among them, the numbers of CD11b^+^CD19^+^ B1 cells and CD11b^+^ myeloid cells were markedly increased, as shown in **Figure 3B**. Given the critical role of B1 cells-secreted IgM in combating bacterial pathogens, we further examined whether the omentum is essential for the production of IgM during peritoneal infections. The PLF samples were collected from WT and omentectomized mice at 8 and 24 h after CLP and the level of IgM was assessed by ELISA. As we expected, the concentration of IgM was significantly lower in omentectomized mice (**Figure 3C**), suggesting that omental B1 cells can serve as an important source of IgM during peritoneal infections. Altogether, these findings revealed that omental MSs were promptly triggered in order to achieve the mobilization of immunological effectors against the bacterial challenge and were indispensable for the generation of protective IgM during peritoneal infections.

### Essential Involvement of the Omentum in Neutrophils Recruitment During CLP-Induced Peritonitis

**Figure 4:**
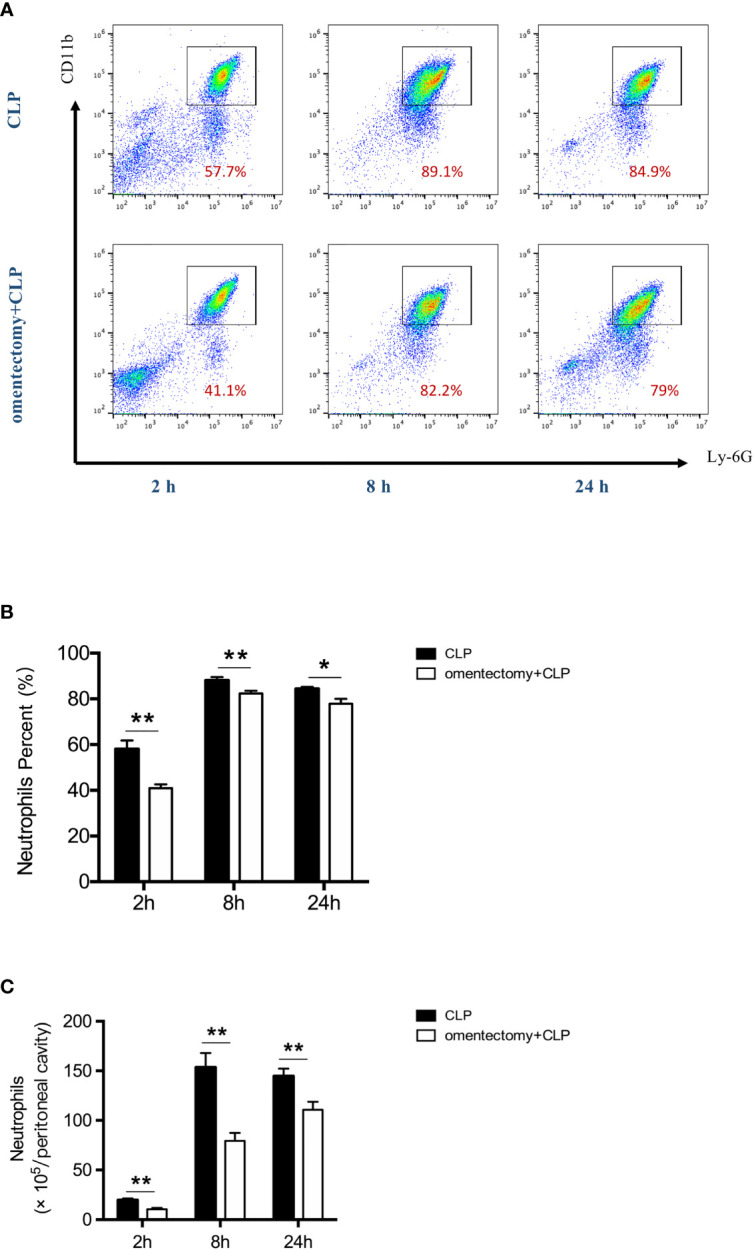
The omentum regulates neutrophils recruitment during CLP-induced peritonitis. At indicated time intervals after CLP, peritoneal cells were collected from WT and omentectomized mice (n = 6 per group). Number indicates the percentage of CD11b and Ly6G double-positive cells, and the percentages **(A, B)** and numbers of neutrophils **(C)** were analyzed by flow cytometry. Results are expressed as means ± SEM. **p < 0.01 and *p < 0.05 for omentectomized vs WT.

The neutrophil is a key component of innate immunity and is required for the survival of sepsis patients (17). Rapid recruitment of neutrophils to the sites of infection is critical for bacterial containment and eradication (18). Thus, to examine the effects of omentectomy on intraperitoneal neutrophil trafficking, we measured the percentages and numbers of neutrophils in the peritoneal cavity during CLP-induced peritonitis. The results demonstrated that the population of CD11b^+^ Ly6G^+^ neutrophils was increased in the peritoneal cavity at the first 2-h time point after CLP (**Figures 4A, B**). However, the percentages and absolute numbers of neutrophils in the peritoneal cavity were significantly lower at 2 h after CLP in the absence of omentum (**Figures 4B, C**). At 24 h after CLP, the percentages and numbers of neutrophils were still lower in omentectomized mice (**Figures 4B, C**). These observations implied that the omentum was indispensable for the substantial recruitment of neutrophils in CLP-induced peritonitis.

### Essential Involvement of the Omentum in Neutrophils Activity During CLP-Induced Peritonitis

**Figure 5:**
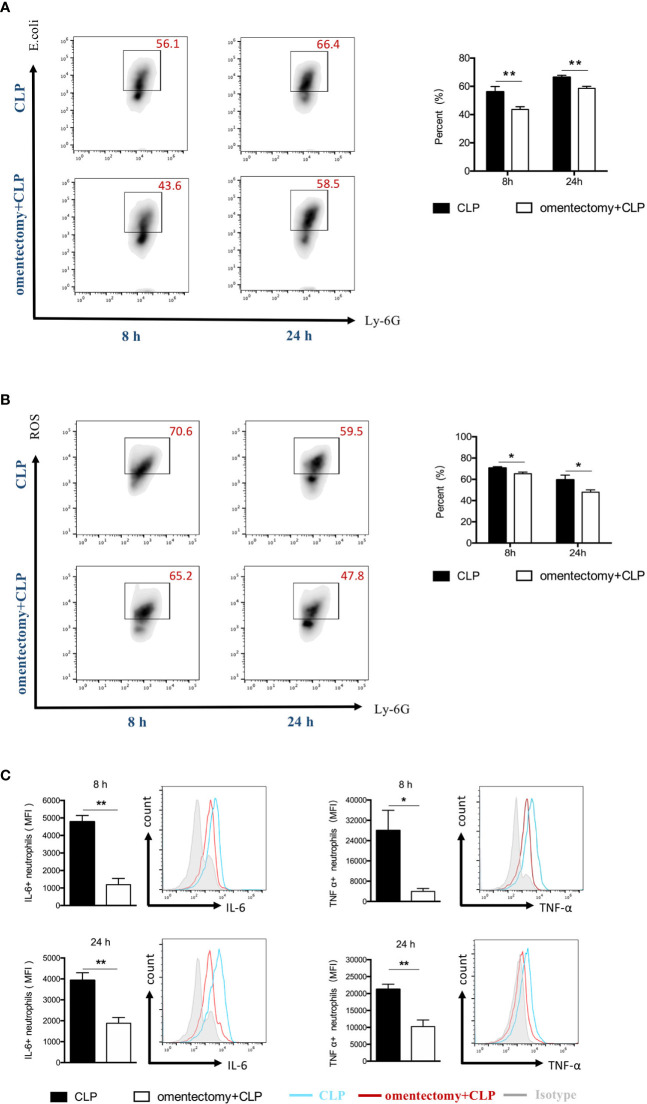
The omentum regulates the functional activity of peritoneal neutrophils during CLP -induced peritonitis. At indicated intervals, peritoneal cells were collected from WT and omentectomized mice (n = 6 per group). Cells were stained for neutrophil markers (Ly6G^+^CD11b^+^) and assessed for phagocytic capacity **(A)** or ROS production **(B)** as described in Materials and Methods. Cells were stained for neutrophil surface markers (Ly6G^+^CD11b^+^) and intracellular cytokine antibodies for TNF-α or IL-6 **(C)**. The bar graph represents the average mean fluorescence intensity (MFI) of TNF-α and IL-6 in neutrophils. Data are representative of three independent experiments with similar results, and data in the bar graph are shown as means ± SEM. **p < 0.01 and *p < 0.05 for omentectomized vs WT.

To effectively eliminate the pathogen, neutrophils are required to phagocytize the bacteria and destroy them after engulfment. It is worth noting that the host cells can release ROS in phagosomes and ultimately eliminate the pathogens (17). In the present study, neutrophils were isolated from the peritoneal cavity, and their phagocytic ability and ROS production were assessed using flow cytometric analysis. The results demonstrated that the phagocytic ability of neutrophils was significantly reduced in omentectomized mice at all time points after CLP compared to WT mice (**Figure 5A**). Similarly, the neutrophils isolated from omentectomized mice had a significant decrease in ROS generation at the indicated intervals after CLP than those isolated from WT mice (**Figure 5B**). Besides, TNF-α and IL-6 are pleiotropic cytokines produced mainly by neutrophils, which participate in cell trafficking, inflammatory responses, and host defense against infection. Then, peritoneal cells were isolated at the indicated intervals after CLP, stained with CD11b and Ly6G, and the levels of TNF-α (**Figure 5C**) and IL-6 (**Figure 5C**) were examined by intracellular cytokine staining. As shown in **Figure 5C**, the peritoneal neutrophils collected from WT or omentectomized mice produced substantial levels of TNF-α and IL-6 in response to CLP procedure. More importantly, TNF-α and IL-6 production in the neutrophils of omentectomized mice was impaired compared to those of WT mice. These findings revealed that, in addition to ineffective recruitment, there were broad functional defects in peritoneal neutrophils after the removal of the omentum during infectious stimulation.

## Discussion

Peritoneal cavity is a peculiar biological compartment, in which immediate immune responses are needed when the integrity of the digestive tract is compromised or lost (1). In this cavity, the omentum contains a band of intra-abdominal fat tissues that run to the duodenal lobe of the pancreas from the distal spleen and comprises of multiple immune cell aggregates called MSs, in which some of the immune cells are rare and unique in other lymphoid organs (19, 20). Recently, the potential roles of the omentum in peritoneal defensive mechanisms have attracted great attention from both clinical and basic researchers around the world (21). Indeed, the omentum is able to move along the peritoneal cavity and occlude at the site of inflammation, including surgical wounds, ulcerated intestine, and inflamed appendix (7). Therefore, more than one century ago, Rutherford Morrison referred to the omentum as “the abdominal policeman” (22). A previous retrospective study has reported that ileoanal anastomosis patients who underwent omentectomy during proctocolectomy tend to have worsened outcomes (23), which implicates a close relationship between omentectomy and higher risks of sepsis in patients (24). In experimental peritonitis, the removal of the omentum has been demonstrated to reduce survival rates and affect local peritoneal defense mechanisms (4). Our results, in agreement with previous findings (4), showed that the mortality rate of mice exposed to omentectomy and CLP was remarkably lower compared to those treated with CLP alone. Furthermore, these mice exhibited impairment in intraperitoneal bacterial clearance, together with an increase in the expression of proinflammatory cytokines after omentectomy and CLP when compared to CLP alone. It should be noted that these observations are contrasted with those published by Wang et al. (4) showing that no differences in bacterial load within the peritoneum or blood after the removal of the omentum. Differences in the severity of CLP-induced peritonitis or the types of mice may be the explanation for this discrepancy. Collectively, our findings implied that the omentum could affect the outcomes of CLP-induced peritonitis, and its removal eventually resulted in the development of greater bacteremia, exaggerated systemic inflammation, and worsen survival.

The MSs in the omentum was first described by the French anatomist Ranvier in 1874, due to their whitish appearance amidst the yellow fat (7). Nowadays, it is well known that MSs are organized aggregates of leukocytes embedded between adipocytes in the omentum (24). Recent findings illustrated that in addition to collect fluids, particulates, and cells, MSs of the omentum can act as a unique secondary lymphoid tissue that enhances immune responses against peritoneal antigens (12). Enhanced maturation and increased number of MSs have been reported to be induced upon exposure to microbiota or microbial products (20). In the present study, our data clearly showed that, in the steady-state, relatively small and few MSs were scattered throughout the omentum; and after CLP procedure, the number and size of MSs were increased tremendously during the first 8 h (12). Moreover, it had been reported that the cell populations in MSs differed markedly from those in typical lymphoid tissues. B1 cells are a specific subset of B cells, which mainly locate in the peritoneal cavity and make up the majority of lymphocytes in MSs. Unlike conventional B (B2) cells, B1 cells are specialized in providing the first wave of IgM antibodies required for bacterial pathogen clearance, which is crucial to combat fast-replicating microorganisms (19, 25). Our results showed that, in resting conditions, MSs were mostly comprised of IgM^+^ B cells, along with low quantities of CD11^b+^ myeloid cells and CD^3+^ T cells. Following CLP exposure, the marked enlargement of MSs and high amount of IgM^+^ cells were observed. Although it is not practical to remove MSs solely and address their role in protection directly, we noticed that, in the absence of MSs upon omentum removal, these mice failed to produce sufficient IgM in the peritoneal cavity and were more susceptible to abdominal infection. A previous study (26) demonstrated that the sepsis mice lacking of IgM after CLP produce exhibited remarkably higher mortality than their WT counterparts, suggesting that IgM confers protection again bacterial elimination from the peritoneal cavity (21). Taken together, these data suggest that MSs are critical inducible secondary lymphoid structures in the omentum that support innate-like B1 cell responses and local IgM production, thereby improving the local immunity during peritoneal infections.

Upon infectious stimulation, neutrophils are the most abundant component of the host immune system that provide the first line of defense against invading pathogens (27). These immune cells are recruited immediately to the site of infection or inflammation, where they can exert antimicrobial functions (28). Neutrophils have been implicated in the control of a variety of bacterial infections and inherited genetic disorders that affect neutrophil function, resulting in profound susceptibility to bacterial infections (29). In septic peritonitis, it is characterized by a massive infiltration of neutrophils into the peritoneal cavity, which is essential for effective bacterial clearance (30). In accordance with previous studies, we showed that, in a steady-state, few neutrophils were observed in the peritoneal cavity, and neutrophil influx could be promptly provoked by abdominal inflammation. However, the influx of peritoneal neutrophils was decreased in omentectomized mice at the indicated time points after CLP surgery. These findings corroborate a recent report about the modulatory effect of the omentum on neutrophil migration in septic peritonitis. Furthermore, Buscher *et al.* (2) suggest that the omentum is involved in neutrophil deployment during peritoneal infections. In addition to successful migration to the site of infection, there are some functionally activated neutrophils in the peritoneal cavity to combat invading pathogens. Neutrophils kill pathogens through different mechanisms: phagocytosis, releasing ROS, proteinase-induced degranulation, etc. Neutrophils also produce several cytokines to recruit and activate other immune cells (31). In this study, we found that removal of omentum also comprised neutrophil functions in the peritoneal cavity, as revealed by impaired phagocytosis, decreased oxidative burst, and downregulated expression of TNF and IL-6, which might lead to impaired peritoneal clearances and increased mortality rates. Altogether, these findings indicate that the omentum is also critical for supporting and coordinating neutrophil responses in the peritoneal cavity during peritonitis.

The inflammation-inducible MSs are the major sites for the occurrence of immune responses in the peritoneal cavity, and the reciprocal interactions between immune cells and fibroblastic reticular cells (FRCs) within MSs could potentially influence their functions (26, 32). Then, it is conceivable that the rapid neo-formation of large quantities of MSs in the omentum may function as a microenvironment for proper neutrophil migration and functions in the peritoneal cavity during peritonitis. As one of the most abundant cell types in MSs, B1 cells can release polyreactive IgM that non-specifically recognizes and neutralizes microbes as well as simultaneously produces immunomodulatory molecules such as granulocyte-monocyte colony-stimulating factor and interleukin-3 (IL-3) (33). The crosstalk effect between B-1 cells and macrophages has been demonstrated in a previous report (34). B1 cells derived-IL-3 is considered a critical driving force of sepsis and is responsible for the proliferation and mobilization of neutrophils and inflammatory monocytes (33). Therefore, there may be a leukocyte communication hierarchy in which B1 cells educate their client myeloid cells, or in other words, B1 cells accumulated in MSs during peritoneal infection may have a direct or indirect impact on neutrophils. Besides, FRCs in MSs have been regarded as an important regulator of both adaptive and innate immunity in responses to microbial invasions by interacting with neighboring immune cells in lymphoid tissues (32). Future studies are needed to elucidate the precise sequence of events that link B1 cell or FRC activation to neutrophil function upon infectious stimulation.

Altogether, protective immune responses at the peritoneal cavity require rapid and sufficient local immunological responses to defend against pathogens and prevent systemic dissemination of infection (35, 36). Accumulating evidence suggests that the omentum harboring MSs in the peritoneal cavity takes on this responsibility to meet these challenges. The current observations verified the protective role of the omentum during septic peritonitis and further demonstrated that the omental MSs are not only the sites of peritoneal B1 cell activation and local IgM production but also crucial for mediating neutrophil activity and migration in responses to infectious challenges. These findings broaden our understanding of the immunological defensive mechanism in the peritoneal cavity. Especially, the omentum contributes more to peritoneal immune responses during septic peritonitis than has been heretofore recognized. Thus, harnessing the function of MSs-harboring omentum to increase its protective effects may have important biological and therapeutic implications for the control of peritoneal infections.

## Data Availability Statement

The original contributions presented in the study are included in the article/Supplementary Material. Further inquiries can be directed to the corresponding authors.

## Ethics Statement

The animal study was reviewed and approved by Scientific Investigation Board of Tianjin Medical University.

## Author Contributions

TM and Y-mY conceived and designed the study. YL, J-nH, NL, JZ and S-cL did laboratory work and data analysis of the experiments. All authors contributed to the article and approved the submitted version.

## Funding

This work was supported by grants from the National Natural Science Foundation of China (Nos. 81871546, 81730057, 81471841), National Key Research and Development Program of China (no. 2017YFC1103302) and the Tianjin Research Innovation Project for Postgraduate Students (no. 2019YJSB102).

## Conflict of Interest

The authors declare that the research was conducted in the absence of any commercial or financial relationships that could be construed as a potential conflict of interest.
